# Strategy for Determining the Stochastic Distance Characteristics of the 2D Laser Scanner Z + F Profiler 9012A with Special Focus on the Close Range

**DOI:** 10.3390/s18072253

**Published:** 2018-07-12

**Authors:** Erik Heinz, Markus Mettenleiter, Heiner Kuhlmann, Christoph Holst

**Affiliations:** 1 Institute of Geodesy and Geoinformation, University of Bonn, Nussallee 17, 53115 Bonn, Germany; heiner.kuhlmann@uni-bonn.de (H.K.); c.holst@igg.uni-bonn.de (C.H.); 2 Zoller & Fröhlich GmbH, Simoniusstraße 22, 88239 Wangen im Allgäu, Germany; m.mettenleiter@zofre.de (M.M.)

**Keywords:** mobile mapping, kinematic laser scanning, distance measurements, precision, intensity, stochastic model, close range optimization, optical efficiency

## Abstract

Kinematic laser scanning with moving platforms has been used for the acquisition of 3D point clouds of our environment for many years. A main application of these mobile systems is the acquisition of the infrastructure, e.g., the road surface and buildings. Regarding this, the distance between laser scanner and object is often notably shorter than 20 m. In the close range, however, divergent incident laser light can lead to a deterioration of the precision of laser scanner distance measurements. In the light of this, we analyze the distance precision of the 2D laser scanner Z + F Profiler 9012A, purpose-built for kinematic applications, in the range of up to 20 m. In accordance with previous studies, a clear dependency between scan rate, intensity of the backscattered laser light and distance precision is evident, which is used to derive intensity-based stochastic models for the sensor. For this purpose, a new approach for 2D laser scanners is proposed that is based on the static scanning of surfaces with different backscatter. The approach is beneficial because the 2D laser scanner is operated in its normal measurement mode, no sophisticated equipment is required and no model assumptions for the scanned surface are made. The analysis reveals a lower precision in the range below 5 m caused by a decreased intensity. However, the Z + F Profiler 9012A is equipped with a special hardware-based close range optimization partially compensating for this. Our investigations show that this optimization works best at a distance of about 2 m. Although increased noise remains a critical factor in the close range, the derived stochastic models are also valid below 5 m.

## 1. Introduction

Laser scanning is one of the established measuring techniques in the geodetic community and its neighboring disciplines. The principle of laser scanning is based on the acquisition of the environment in the form of 3D point clouds. Several reviews [1,2,3,4] provide an insight into the metrology and its applications. In addition to static laser scanning, kinematic laser scanning with moving platforms has also been subject to continuous developments in recent years. In kinematic laser scanning, typically 2D laser scanners are applied that record 2D scanning profiles of the object space. The third dimension is generated by moving the 2D laser scanner on a mobile platform (e.g., car, train, trolley or person) through the environment. By combining the individual 2D scanning profiles along the travelled path, a 3D point cloud can be obtained. Simultaneously, the trajectory of the 2D laser scanner has to be determined in order to register the scan points in a superior coordinate frame. This can be realized by means of additional sensors (e.g., Global Navigation Satellite Systems GNSS, Inertial Measurement Units IMUs or odometers) or information from the object space provided by the recording sensors (e.g., laser scanners or cameras). More details are given in the reviews of [1,5,6,7]. The growing acceptance of kinematic laser scanning is clearly demonstrated by the increasing number of commercially available systems. Five years ago, Puente et al. [5] provided a review of current mobile mapping and surveying technologies as well as associated systems. However, due to the high dynamics in this field, this review is no longer up to date from today’s perspective.

Kinematic laser scanning systems are frequently used to acquire road corridors [6,8,9,10] or entire city areas [11]. These data can be utilized for maintenance purposes, project planning, modeling or reconstruction. Objects of interest are not only the road surface, but also road markings, road signs, sidewalks, power lines and structures like bridges or tunnels. In addition to pure road inventory, there are more challenging applications like monitoring of the road surface [12] or determination of accurate geometric parameters of roads [13,14] and rail tracks [15,16]. Moreover, measurements in tunnels [13,14], where the systems are faced with GNSS blocking, are noteworthy. Another important application of kinematic laser scanning systems is the determination of clearance gauges. This is crucial for both road traffic [6,8,13] and rail traffic [15,16,17] to ensure that vehicles do not collide with obstacles such as bridges, tunnel walls, road signs, power lines, catenaries or vegetation.

In all mentioned applications, operators are faced with short measuring distances, often notably shorter than 20 m. The laser scanners are typically mounted a few meters above the ground and objects of interest such as buildings, tunnels or bridges are also located close to the route. The same situation is given for indoor systems on trolley platforms [18,19] or carried by a person [20,21] with distances being mostly shorter than 5 m. Despite the improvements in laser scanning technology leading to a variety of sensors [5,9,22], measurements in the close range of several meters around the laser scanner remain critical. Inherently, the focus of the optical receiving unit of laser scanners used for surveying is set to infinity. Hence, at medium and larger distances, parallel incident laser light leads to a good optical efficiency resulting in a sufficient signal input power. In contrast, at shorter distances, divergent incident laser light leads to a defocusing of the optical receiving unit and, thus, to a reduction of the signal input power [23]. This can considerably decrease the intensity of the backscattered laser light in the close range. The studies of [24,25,26] indicate that the precision of distance measurements strongly depends on the intensity of the backscattered laser light: the lower the intensity, the bigger the noise. As a consequence, the defocusing of the optical receiving unit in the close range can deteriorate the precision of the distance measurements. This is also critical because the surfaces in the close range are often very dark, e.g., the road surface or walls in old sooted railway tunnels.

In the light of this, we address the intensity-dependent stochastic distance characteristics of 2D laser scanners with special focus on the close range. This is where the sensors tend to be less precise due to the physical properties of the backscattered laser light. 2D laser scanners as used in kinematic applications are of special interest since the relevant information is often located in the critical close range. In this contribution, we focus on the examination of the Z + F Profiler 9012A [27]. Regarding the close range, the Z + F Profiler 9012A is of particular interest because it is equipped with a special hardware optimization. This optimization reduces the measurement noise in the range between 1 m and 5 m by improving the intensity of the backscattered laser light. Against this background, the main scientific contributions of this work are twofold:

1. This study proposes a new general approach for determining intensity-based stochastic models for 2D laser scanners. This is vital for our investigations because previous research [24,25,26] only addressed 3D laser scanners. Based on the static scanning of surfaces with different backscatter, our approach is beneficial because the 2D laser scanner is operated in its normal measurement mode, no model assumptions for the scanned surface are made and no sophisticated equipment is required. Therefore, the approach can also be applied by end users.
2. The approach is used to derive three intensity-based stochastic models for the three scan rates of the Z + F Profiler 9012A. These models describe the precision of the sensor in general. Moreover, the approach is used for investigating the sensor’s behavior in the range of up to 20 m. In this context, the performance of the hardware-based close range optimization of the Z + F Profiler 9012A is analyzed. As a result, an increased noise in the range below 5 m is verified caused by a decreased intensity but not contradicting the general stochastic models. The sensor’s close range optimization partially compensates for this performing best at about 2 m.

## 2. Methodologies for Quality Assessment of Laser Scanners

Laser scanners have been used for surveying for almost 20 years. In parallel, their accuracy and precision, which we refer to as quality, have been investigated frequently. According to [28,29], the factors influencing the quality of laser scanner point clouds can be classified into four categories: mechanics of the instrument, atmospheric and environmental conditions, object properties as well as scanning geometry. While the quality of the angular measurements only depends on the mechanics of the instrument, all four categories influence the distance measurements. Although the atmospheric and environmental conditions can normally be neglected or sufficiently be corrected [30], the quality of a measured distance is still a cumulative effect of the remaining factors. In the following, we will only address the distance measurements.

The accuracy of a measured distance is influenced by systematic errors that can be calibrated if caused by the instrument [30]. However, uncalibrated instrumental errors as well as object properties and the scanning geometry can corrupt the accuracy. Therefore, many publications have analyzed the accuracy of laser scanners, e.g., by comparing their measurements with reference values provided by an interferometer [2,13,31,32], a total station [33] or a theodolite measurement system [34]. Alternatively, it is also possible to use target-based test fields with or without reference information [31,35,36,37,38] as well as test objects [33,35,38]. An overview can be found in [30].

The precision of distance measurements is defined by random errors. In the majority of cases, the precision is analyzed by scanning and approximating geometric primitives like planes [28,31,35,39,40] or spheres [33,35]. The residuals allow for a quantification of the noise. According to [24], however, this strategy has several drawbacks: The determined noise is a mixture of both angular and distance-related errors. In addition, the scan resolution and the redundancy play an essential part for the approximation, which also depends on the used software. The most important drawback is that the approach relies on the quality of the geometric primitives that has to be significantly better than the noise of the laser scanner. As a solution, Wujanz et al. [24] turn off the rotation of the deflecting mirror and operate the laser scanner in a 1D measurement mode. Thus, the noise can be determined by multiple measurements of an identical point. This methodology is also applied in [2,41].

Investigations of the accuracy and precision of laser scanners are usually carried out for different distances. In addition, the influence of different incidence angles of the laser beam is analyzed in many studies [13,28,33,34,35,39,40]. Both distance and incidence angle represent the influence of the scanning geometry. Moreover, a variety of studies addresses different object properties like color, material or roughness [13,31,32,35,39,40,42,43]. However, universal statements about the quality of laser scanner measurements can hardly be made based on these investigations.

Many studies indicate the relation between measurement noise and backscattered intensity. This is confirmed by the laser-radar equation [44] that links the backscattered intensity to the instrumental characteristics, environmental conditions, scanning geometry as well as object properties. Recently, Wujanz et al. [24] analyzed this in more detail and succeeded in determining an intensity-based stochastic model for the distance measurements of a 3D laser scanner. This was realized by repeated measurements of an identical point in the 1D measurement mode of the sensor for different distances, incidence angles and object properties. The authors found an exponential functional relationship between distance noise and backscattered intensity. This means that the influence of scanning geometry and object properties on the precision of distance measurements can be encoded by the backscattered intensity. A major drawback of the methodology in [24] is that the laser scanner is operated in a 1D mode. The 1D mode is not available to users due to the bundled energy of the laser, which is extremely safety-critical. As a solution, modified approaches were proposed in [25,26], where planar targets were scanned in the normal 3D measurement mode of the laser scanner in order to derive the noise from the residuals of an approximation. This noise is linked to the target’s mean intensity. However, this approach requires model assumptions. Moreover, problems could arise if the targets have irregular backscatter.

The review of the literature demonstrates that the quantification of the distance precision using the intensity of the backscattered laser light seems to be promising. However, previous studies have only focused on 3D laser scanners measuring in 1D or 3D mode [24,25,26], while this study aims at analyzing the precision of a 2D laser scanner. Hence, we propose a new general approach for determining an intensity-based stochastic model of a 2D laser scanner. The approach is beneficial since it is feasible for end users. This is achieved by operating the laser scanner in its normal 2D measurement mode as well as omitting sophisticated equipment and model assumptions for the scanned surface. Moreover, the new approach serves as a basis for a more detailed investigation of the close range. In this regard, the special hardware optimization of the Z + F Profiler 9012A for measurements in the range between 1 m and 5 m is of particular interest [27]. The close range is critical for many kinematic applications and has not been sufficiently investigated so far.

## 3. Characteristics of the 2D Laser Scanner Z + F Profiler 9012A

In this section, the investigated 2D laser scanner Z + F Profiler 9012A is introduced. In Section 3.1, the working principle is presented. This is followed by an explanation of the specifications for accuracy and precision in Section 3.2. This is important to interpret the results of our investigations. Section 3.3 gives the theory about distance measurements in the close range and it explains how the laser scanner’s close range optimization aims at improving the measurement precision.

### 3.1. Construction of the Z + F Profiler 9012A

**Figure 1:**
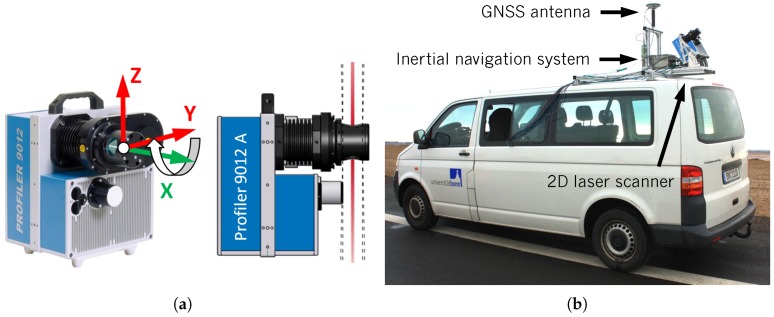
(**a**) Z + F Profiler 9012A with 2D scanning profile in red (YZ-plane) and rotational axis of the deflecting mirror in green (X-axis) [27]; (**b**) kinematic laser scanning system [45] equipped with the Z + F Profiler 9012A and an inertial navigation system iMAR iNAV-FJI-LSURV [46].

The Z + F Profiler 9012A (Figure 1a) is a 2D laser scanner manufactured by Zoller & Fröhlich GmbH (Wangen im Allgäu, Germany) and purpose-built for kinematic applications [27]. At the University of Bonn, the Z + F Profiler 9012A is part of a kinematic laser scanning system (Figure 1b) acquiring 3D point clouds [45]. In addition, the kinematic laser scanning system is equipped with an inertial navigation system iMAR iNAV-FJI-LSURV consisting of three fiber-optic gyroscopes, three servo accelerometers and an RTK-GNSS [46].

The Z + F Profiler 9012A has a field of view of 360${}^{\circ }$ within its YZ-plane, i.e., the deflecting mirror rotates about the X-axis of the sensor (Figure 1a). Hence, a measured point $x_{j}$ is given by:

(1) $$x_{j}=\left[\begin{array}{c} x_{j}\\ y_{j}\\ z_{j} \end{array}\right]=\left[\begin{array}{c} r_{j}\cdot \sin\theta_{j}\cdot \sin\varphi_{j}\\ r_{j}\cdot \sin\theta_{j}\cdot \cos\varphi_{j}\\ r_{j}\cdot \cos\theta_{j} \end{array}\right]\approx \left[\begin{array}{c} r_{j}\cdot \sin\theta_{j}\cdot \sin0^{\circ }\\ r_{j}\cdot \sin\theta_{j}\cdot \cos0^{\circ }\\ r_{j}\cdot \cos\theta_{j} \end{array}\right]\approx \left[\begin{array}{c} 0\\ r_{j}\cdot \sin\theta_{j}\\ r_{j}\cdot \cos\theta_{j} \end{array}\right],$$

where $x_{j}$, $y_{j}$ and $z_{j}$ denote Cartesian coordinates. The related polar coordinates, which correspond to the original measurements of the laser scanner, are given by distance $r_{j}$, horizontal direction $\varphi_{j}$ and vertical angle $\theta_{j}$ within the 2D scanning profile. In theory, the horizontal direction $\varphi_{j}$ should be 0${}^{\circ }$. However, due to the imperfectness of the beam deflection unit of the sensor, $\varphi_{j}$ is not exactly 0${}^{\circ }$. This error is calibrated by the manufacturer, i.e., $x_{j}$ is only approximately zero.

The speed of the deflecting mirror can be set to 50 rps, 100 rps and 200 rps (rps = rotations per second). In addition, the resolution can be set to 5120, 10,240 or 20,480 points per scanning profile. The combination of both rotational speed of the deflecting mirror and point resolution leads to different scan rates of 254 kHz, 508 kHz or 1016 kHz. Due to the fact that the maximum scan rate is limited to 1016 kHz, six combinations are selectable (Table 1). The distance measurement works on the principle of phase shift and has an operating range between 0.3 m and 119 m [27].

### 3.2. Specifications for the Distance Measurements of the Z + F Profiler 9012A

The data sheet of the Z + F Profiler 9012A provides specifications for the accuracy and the precision of the distance measurements [27]. For the accuracy, range drifts of < 2 mm (without internal reference) and < 0.3 mm (with internal reference) are specified. Furthermore, an error from linearity of < 1 mm is stated, which indicates the maximum expected error between the measured distance and the true distance. The determination of this value is realized by the comparison of measured distances with interferometric reference distances during the sensor calibration process. For this purpose, distance measurements are performed in the 1D mode of the laser scanner [2,41].

The distance precision primarily depends on the object properties, the scanning geometry and the scan rate. As shown in [24], it is possible to encode the influence of scanning geometry and object properties by the backscattered intensity. Based on this, a stochastic model for each scan rate of the laser scanner can be derived describing the distance noise as a function of intensity. Instead of using intensities, the specifications of the Z + F Profiler 9012A (Table 2) refer to an incidence angle of 0${}^{\circ }$ at varying distances (scanning geometry) and reflectivities (object properties). The noise of the distance measurements is determined for each specific combination by means of repeated measurements of an identical point in the 1D measurement mode of the laser scanner [2,41].

Table 2 lists the related standard deviations $\sigma_{r}$ of the distance measurements for a standard scan rate of 127 kHz. Since these values are only valid for a scan rate of 127 kHz, they have to be converted to the actual scan rates of 254 kHz, 508 kHz or 1016 kHz. The conversion factors of 1.4, 2.0 and 2.8 (Table 1) can be derived from the law of error propagation for uncorrelated observations with equal precision [47]. This is explained using an example: when measuring with a scan rate of 1016 kHz, the laser scanner records 1,016,000 points per second. By reducing the scan rate to 127 kHz, i.e., one-eighth of 1016 kHz, eight consecutive measurements within the scanning profile are averaged to a single value [2]. Thus, in theory, the noise is reduced by a factor of $\sqrt{8}\approx 2.8$.

### 3.3. Close Range Optimization of the Z + F Profiler 9012A

**Figure 2:**
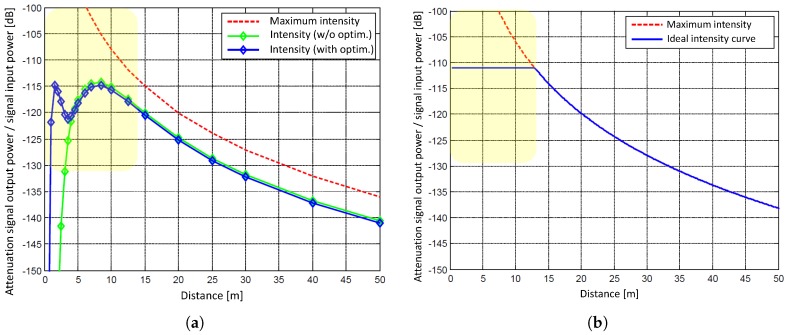
(**a**) Simulation of the optical receiving unit of the Z + F Profiler 9012/9012A (without/with close range optimization) as a function of the distance between laser scanner and object. (**b**) Simulation of an ideal intensity curve (by courtesy of Zoller & Fröhlich GmbH, Wangen im Allgäu, Germany).

According to the manufacturer, the Z + F Profiler 9012A is equipped with a special optimization of the hardware improving the precision of distance measurements in the range between 1 m and 5 m [27]. The reduction of the noise is achieved by increasing the signal input power and, hence, the intensity of the backscattered laser light in the close range. This is illustrated in Figure 2. Please note that the curves in Figure 2a apply to the Z + F Profiler 9012 (green, without close range optimization) and Z + F Profiler 9012A (blue, with close range optimization) and might be different for other laser scanners, though the general behavior should be comparable [23].

In Figure 2a, the red dotted curve indicates the intensity of the backscattered laser light for a system with an optical efficiency of 100 %. The curve follows the signal attenuation with the squared distance as expected from the radar-laser equation [44] leading to a distance-related noise of the measurements. This would result in an extremely high intensity directly in front of the sensor. However, the red curve represents the physical limit that is not reached in reality. In reality, the green curve is obtained. The green curve follows the behavior of the red curve with a certain offset caused by inevitable losses during the measurement process due to the instrument, the atmosphere, the scanning geometry or the object properties. In the close range, however, the green curve suddenly drops after having reached its maximum value at about 8 m. This leads to a smaller signal input power at 2.5 m than at 50 m and, as a consequence, to a poor precision of the measurements in the close range.

The abrupt drop of the backscattered intensity in the close range is caused by an interference of the increase due to the squared distance with two other effects:

1. The focus of the optical receiving unit of the laser scanner is set to infinity, i.e., at medium and larger distances, parallel incident laser light leads to a good optical efficiency resulting in a sufficient signal input power. In contrast, in the close range, divergent incident laser light leads to a defocusing of the received laser spot on the avalanche photodiode (APD). This means that not all of the light received by the optics actually falls on the APD and, thus, is converted into an electrical signal. The intensity maximum at about 8 m arises from the fact that for distances smaller than 8 m more light is lost by the defocusing than is collected by the optical aperture due to the increase by the squared distance.
2. The optical receiving unit is constructed as a coaxial system. The received laser beam is coaxially guided around the emitted laser beam. Thus, the aperture for the emitted laser beam is located exactly in the center of the received laser beam. This aperture is like a shadow in the cross section of the received laser beam as seen from the APD. At short distances, this shadow grows larger and at some point covers the APD completely. In Figure 2a, this is indicated by the abrupt drop of the intensity curve for distances smaller than 4.5 m.

A discussion about this can also be found in [23]. Furthermore, Zámečníková et al. [32] empirically derive intensity curves showing the behavior of the green curve in Figure 2a.

Low measurement noise has the drawback that a high signal input power has to be applied to the optical receiving unit. However, for signal processing, a small dynamic range is required, meaning that the ideal intensity curve should follow the blue line in Figure 2b. The solution is a modification of the hardware in such a way that laser light can reach the APD in the close range, even though the APD is basically completely shadowed. For this purpose, only a small part of the optical receiving unit is used in order to slightly improve the optical efficiency in the close range; small enough not to influence the optical efficiency for larger distances. This small improvement is sufficient to transform the green curve into the blue curve (Figure 2a). As can be seen in Figure 2a, the optimization performs best at about 2 m before it drops again for distances shorter than 1 m.

In Table 2, the noise specifications for both Z + F Profiler 9012 and 9012A (without and with close range optimization) are stated. It can be seen that the precision can be improved by up to 50 % for the distances of 1 m, 2 m and 5 m when using the close range optimization. The improvement due to the close range optimization is evaluated and verified in Section 6.2. In addition, it is proved that the derived stochastic models (Section 6.1) are valid for both the range below and above 5 m.

## 4. Measurement Concept

In Section 4.1, the measurement concept for the determination of the intensity-based stochastic models and the examination of the close range optimization are presented. Section 4.2 demonstrates that angular errors of the sensor can be neglected when applying our measurement concept.

### 4.1. Experiments

**Figure 3:**
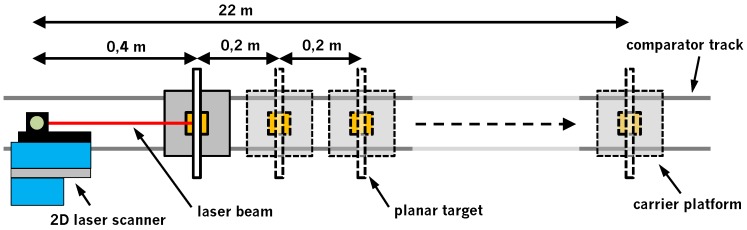
Setup for the investigation of the precision of the distance measurements of the Z + F Profiler 9012A on a railbound comparator track by scanning planar targets at different distances.

**Figure 4:**
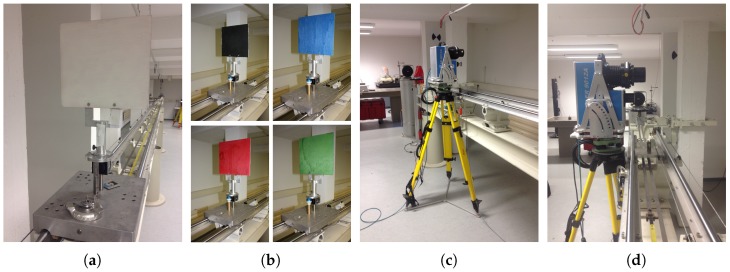
(**a**,**b**): white and colored planar targets that were scanned with the Z + F Profiler 9012A; (**c**,**d**): Z + F Profiler 9012A on a tripod next to the railbound comparator track.

The measurement concept for the investigation of the Z + F Profiler 9012A is illustrated in Figure 3. The setup makes use of a railbound comparator track with a length of 22 m. On this track, a carrier platform can be mounted. In addition, the track contains an integrated interferometer, which allows the carrier platform to be moved in fixed increments using a stepping motor. For the experiments, a white planar target with good backscatter and a size of 0.25 m × 0.25 m was attached to the carrier platform (Figure 4a). For further experiments, the white target was replaced by colored targets (black, blue, red, green) of the same size (Figure 4b). At the end of the track, the Z + F Profiler 9012A was put on a tripod (Figure 4c) and manually aligned in such a way that the scanning profile was approximately parallel to the track (Figure 4d). Thus, the scanning profile hit the target nearly perpendicular (Figure 3). All experiments were performed in the normal 2D measurement mode of the laser scanner.

Based on this setup, different experiments were carried out. In the first experiment, the white planar target was placed at a distance of 0.4 m in front of the 2D laser scanner. Following this, the planar target was moved along the comparator track in increments of 0.2 m up to a distance of 10 m and beyond in increments of 1 m up to a distance of 22 m. For each of the 61 target positions, a static scan with a duration of approximately 30 seconds was executed. As a result, several hundred overlapping profiles were generated for each target position, which allow for a statistic analysis of the noise. The procedure was repeated for all six settings of the Z + F Profiler 9012A (Table 1). This experiment allows for an analysis of the precision and intensity with respect to the distance.

In order to change the scanning geometry with respect to both distance and incidence angle, a second experiment was performed. In this regard, the white target was scanned at different distances (3 m, 5 m, 10 m, 20 m) with incidence angles between 0${}^{\circ }$ and 80${}^{\circ }$. This was realized by rotating the targets around their vertical axis. To include the influence of different object properties, planar targets with different colors (black, blue, red, green) were scanned in a third experiment. This experiment was carried out over the entire range of the track with the scanning profile hitting the targets again nearly perpendicular (cf. experiment 1). The colors were selected to cover the entire spectral range of the intensities as much as possible. Please note that, for time reasons, experiments 2 and 3 were only performed with one setting of the laser scanner (50 rps mirror speed, 1016 kHz scan rate).

A great advantage of the experiments is that they basically do not require sophisticated equipment. The targets are basic wooden boards painted with customary paint and also the comparator track is dispensable because the targets could also be attached to tripods. Thus, the experiments are feasible for every operator. Furthermore, the normal 2D measurement mode is used so that the laser scanner remains a laser protection class 1 device. No special precautions have to be taken.

**Figure 5:**
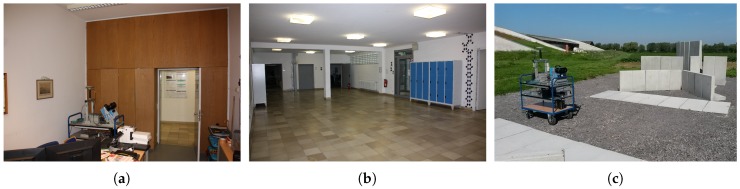
(**a**) Office room, (**b**) hallway and (**c**) concrete blocks in an outdoor environment, scanned with scan rates of 254 kHz and 508 kHz.

Our experiments allow for an analysis of the close range optimization and the determination of intensity-based stochastic models for the distance measurements (cf. Section 5 and Section 6). However, the measurements on the track are time consuming. Hence, experiments 2 and 3 were only performed with a scan rate of 1016 kHz at a mirror speed of 50 rps. In order to derive intensity-based stochastic models for the scan rates of 254 kHz and 508 kHz with less effort, several scans in ordinary indoor and outdoor environments were carried out with the corresponding settings (Figure 5). This confirms that the experiments can be easily performed without supplementary equipment and in normal use outside the laboratory. The environment only needs to include areas producing high and low intensities.

### 4.2. Impact of Angular Errors on the Experiments

**Figure 6:**
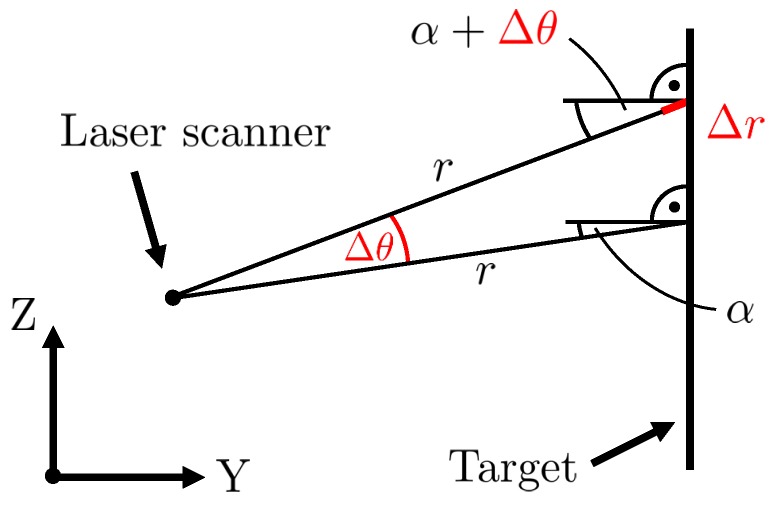
Estimation of the impact Δr on the measured distance *r* caused by an angular error Δθ as a function of the incidence angle α and the distance *r* between target and laser scanner.

The goal of this study is the investigation of the distance precision. Hence, angular errors must not interfere with the results. For this reason, an estimation of the impact of angular errors on the measured distance has been performed. This was carried out based on the measurement concept (Section 4.1) and the manufacturer information for the angular accuracy of the vertical angles, i.e., $\Delta \theta =0.02^{\circ }$ [27]. As can be seen in Figure 6, due to an angular error Δθ, the distance *r* is measured as too long by Δr. The impact Δr depends on three variables, i.e., the angular accuracy Δθ, the incidence angle α and the distance *r*. According to the sine theorem, the impact Δr can be calculated as follows:

(2) $$\frac{r+\Delta r}{\sin(90^{\circ }+\alpha )}=\frac{r}{\sin(90^{\circ }-\alpha -\Delta \theta )}\;\;\Leftrightarrow \;\;\Delta r=\frac{r\cdot \sin(90^{\circ }+\alpha )}{\sin(90^{\circ }-\alpha -\Delta \theta )}-r.$$

During the experiments on the comparator track, the distances between laser scanner and target were between 0.4 m and 22 m. In addition, the targets were mounted at the height of the sensor’s tilting axis. Due to the target size of 0.25 m, the incidence angles in the vertical direction were < 18${}^{\circ }$. The maximum incidence angle is reached at the edge of the targets directly in front of the laser scanner. Using Equation (2), the impact Δr can be calculated for every combination of distance *r* and incidence angle α. The analysis showed that the impact Δr does not exceed 0.045 mm. This value corresponds to approximately 1/2 digit of the sensor’s distance resolution of 0.1 mm [27], meaning that the measured distance changes by 0.1 mm in the most unfavorable case. However, this is below the expected noise, which is in the range of mm to some tenth of a mm (Table 2). Consequently, the impact of angular errors on the results can practically be neglected in this case.

Please note that only the incidence angle in the vertical direction, i.e., within the scanning profile, is relevant. High incidence angles in the horizontal direction as generated by rotating the targets around their vertical axis do not corrupt the results since the horizontal angle φ is assumed to be constant for the entire scanning profile. This requires a stable rotation of the mirror. However, the impact Δr grows for larger distances in combination with larger incidence angles in the vertical direction. This means that certain parts of the ceiling or the ground in the office and hallway should not be used in the analysis. An estimation is feasible using Equation (2).

## 5. Data Analysis

**Figure 7:**
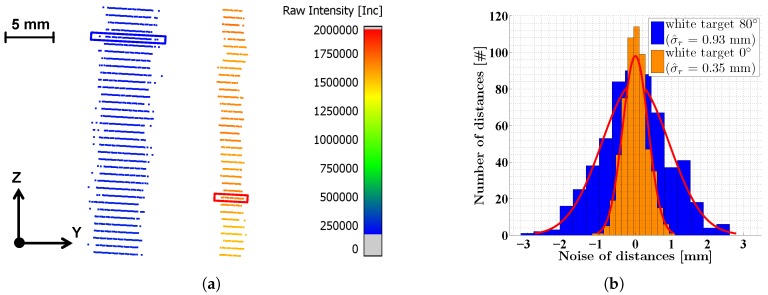
(**a**) two exemplaric scans of the white planar target at a distance of 5 m with an incidence angle of 0${}^{\circ }$ (orange) and 80${}^{\circ }$ (blue), color-coded with the related intensities; (**b**) histograms showing the noise of the distance measurement for two arbitrary angular steps marked with boxes in (**a**).

The data analysis comprises the quantification of the distance precision of the Z + F Profiler 9012A as a function of the backscattered intensity as well as the investigation of the close range optimization. All scans were performed in a static manner for several seconds (Section 4.1). As a result, each scan consists of several hundred overlapping scanning profiles, which can be used to analyze the noise. Figure 7a shows two exemplaric scans of the white planar target at a distance of 5 m measured with an incidence angle of 80${}^{\circ }$ (blue) and 0${}^{\circ }$ (orange). In both scans, the discrete angular steps of the Z + F Profiler 9012A can be distinguished as lines superimposed upon each other. The length of these lines represents the distance noise. Each point is color-coded with its intensity. Please note that the lines with an incidence angle of 80${}^{\circ }$ (blue) show a lower intensity and a higher noise than the lines with an incidence angle of 0${}^{\circ }$ (orange). This can also be seen in Figure 7b showing the normally distributed distance measurements for two randomly selected angular steps (marked with boxes in Figure 7a).

Each target scan comprises *i* angular steps, for which each in turn consists of *n* measured points $x_{j}^{\left(i\right)}$ with j=1⋯n. The analysis is based on the transformation of the Cartesian coordinates *x*, *y* and *z* from Equation (1) into polar coordinates, i.e., distance *r*, horizontal direction φ and vertical angle θ:

(3) $$\left[\begin{array}{c} r_{j}^{\left(i\right)}\\ \varphi_{j}^{\left(i\right)}\\ \theta_{j}^{\left(i\right)} \end{array}\right]=\left[\begin{array}{c} \sqrt{{x_{j}^{\left(i\right)}}^{2}+{y_{j}^{\left(i\right)}}^{2}+{z_{j}^{\left(i\right)}}^{2}}\\ \arctan\left(\frac{x_{j}^{\left(i\right)}}{y_{j}^{\left(i\right)}}\right)\\ \arctan\left(\frac{\sqrt{{x_{j}^{\left(i\right)}}^{2}+{y_{j}^{\left(i\right)}}^{2}}}{z_{j}^{\left(i\right)}}\right) \end{array}\right].$$

In the case of the Z + F Profiler 9012A, polar coordinates could directly be exported from the scanner making the transformation dispensable. Based on Equation (3), the points of each target scan can be sorted by their vertical angles into *i* groups (one for each angular step). For each angular step, the standard deviation ${\hat{\sigma }}_{r}$ of the related distance measurements is calculated indicating the precision:

(4) $${\hat{\sigma }}_{r}^{\left(i\right)}=\sqrt{\frac{\sum_{j=1}^{n}\left(r_{j}^{\left(i\right)}-\frac{\sum_{j=1}^{n}r_{j}^{\left(i\right)}}{n}\right)}{n-1}}.$$

The Gaussian shape of the histograms in Figure 7b justifies the calculation of a standard deviation for the distance measurements. Besides the standard deviation ${\hat{\sigma }}_{r}$, a corresponding mean intensity $\hat{I}$ can be derived for each group:

(5) $${\hat{I}}^{\left(i\right)}=\frac{\sum_{j=1}^{n}I_{j}^{\left(i\right)}}{n}.$$

Please note that unprocessed raw intensities should be utilized. Manufacturers often provide normalized intensities that compensate for the signal attenuation with the squared distance between laser scanner and object [2,44] as well as the transfer function of the optical system in the close range. The reason for this is a better visualization of the results displaying surfaces of the same reflectivity equally. Furthermore, histogram equalization is often performed [2]. However, normalized intensities are not necessarily connected to the signal input power and the distance noise.

By analyzing all target scans in this way, thousands of pairs between standard deviation ${\hat{\sigma }}_{r}$ and mean intensity $\hat{I}$ can be generated. As shown in [24], these data can be used to derive intensity-based stochastic models for the laser scanner describing the precision of the distance measurements as a function of the backscattered intensity. The derivation of such models for the Z + F Profiler 9012A is explicitly carried out in Section 6.1.

Although based on [24], our way of data analysis represents a new method for the examination of the distance precision of 2D laser scanners that became necessary because previous research only addressed 3D laser scanners. In [24], the laser scanner was operated in a 1D measurement mode, being beneficial since it allows for repeated measurements of an identical point. At first glance, this strategy is also applicable to 2D laser scanners. However, the decisive drawback is that the 1D mode is not available to users. The reason is that the illumination of a single point with a fixed laser beam is extremely safety-critical due to the bundled energy of the laser. Our approach using a quasi 1D mode is not safety-critical and operates the laser scanner in its normal measurement mode. Moreover, no sophisticated equipment is required making the approach feasible for end users. In addition, the approach is independent of model assumptions for the scanned surface such as made in the modified approaches. For instance, in [25,26], the noise was calculated from plane residuals and linked to the plane’s mean intensity. However, it must be kept in mind that these approaches were designed for 3D laser scanners and not for 2D laser scanners.

The advantage of working without model assumptions becomes clear from Figure 7a. Especially in the scan with an incidence angle of 0${}^{\circ }$, the non-planarity of the target is clearly visible. Since only the individual angular steps and not the entire target is utilized, this non-planarity can be ignored. This is also confirmed by the normally distributed histograms in Figure 7b. Furthermore, a mean intensity value can be assigned to each angular step instead of using a single intensity value for the entire target when approximating it with a model. Please note that the reflective properties of a scanned target are not necessarily constant for every part of the surface. This is actually the case for the targets depicted in Figure 5. A mean value for the entire target could distort the result. The only disadvantage of the quasi 1D mode is that angular errors might affect the results. However, the estimation in Section 4.2 shows that this is not critical when taking certain constraints for the scanning geometry into account.

## 6. Results and Discussion

In the following, the results of the experiments are presented and discussed. In Section 6.1, the relationship between the intensity of the backscattered laser light and the precision of the distance measurements is examined leading to three intensity-based stochastic models for the three scan rates of the Z + F Profiler 9012A (i.e., 254 kHz, 508 kHz and 1016 kHz). Section 6.2 analyzes the performance of the close range optimization and connects this to the theory from Section 3.3.

### 6.1. Determination of Intensity-Based Stochastic Models

As described in Section 4.1, scans with all six settings of the Z + F Profiler 9012A were performed. These six settings comprise scan rates of 254 kHz, 508 kHz and 1016 kHz (Table 1). In order to derive an intensity-based stochastic model for each scan rate, all pairs of standard deviations ${\hat{\sigma }}_{r}$ and mean intensity $\hat{I}$ (Equations (4) and (5)) from all experiments were pooled for the respective scan rate, whereas, in experiments 1–3, planar targets were scanned on the comparator track, in experiment 4, normal environments were scanned. For the latter, appropriate surfaces were manually extracted (e.g., walls, ground, ceiling). These surfaces were analyzed in the same way as the planar targets on the comparator track. This leads to an extensive data basis (Table 3).

**Figure 8:**
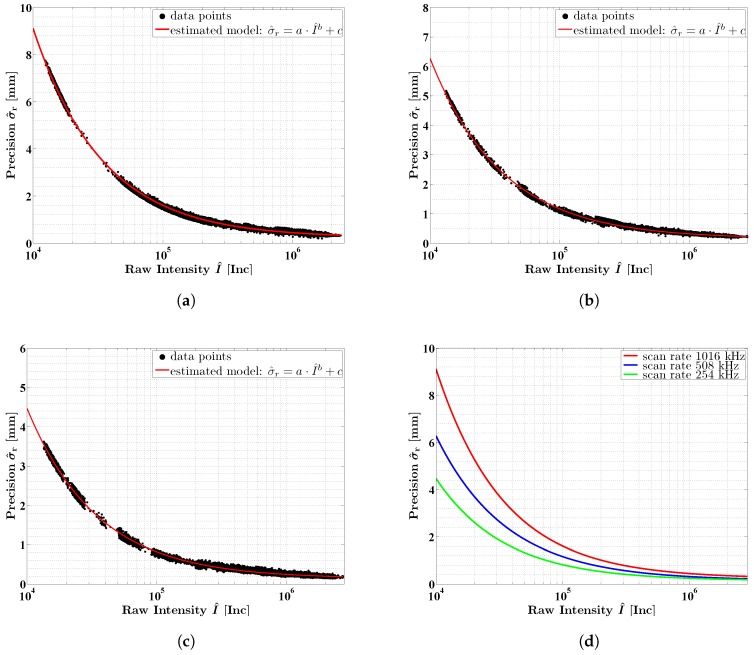
Intensity-based stochastic models for the precision of the distance measurements of the Z + F Profiler 9012A for different scan rates: (**a**) 1016 kHz; (**b**) 508 kHz; (**c**) 254 kHz; (**d**) all models.

Figure 8a–c show the standard deviations ${\hat{\sigma }}_{r}$ of the distance measurements as a function of the mean intensities $\hat{I}$ for all three scan rates. Please note that the horizontal axis is plotted logarithmically. Clearly, all three curves show a similar behavior that applies to an exponential function. For lower intensities, higher standard deviations are obtained. Furthermore, a dependency on the scan rate is apparent: the higher the scan rate, the higher the standard deviation. However, all curves rapidly decrease in the interval between 0 Inc and 500,000 Inc. For higher intensities, the precision only slightly improves and it seems reasonable to assume that the estimated curves continue in the same way.

In the course of experiments 1–3, measurements with a scan rate of 1016 kHz were carried out on both white and colored targets as well as targets with varying incidence angles. In addition to it, the measurements covered a range between 0.4 m and 22 m. Yet, all of these different measurements with respect to object properties and scanning geometry basically follow the exponential behavior of the curve. The black and green targets produced the lowest intensities, whereas the white, blue and red targets produced higher intensities. The measurements under different incidence angles between 0${}^{\circ }$ and 80${}^{\circ }$ lead to very different intensities decreasing with higher incidence angles. The dependency on the distance is discussed in more detail in Section 6.2.

Please note that the measurements in the normal indoor and outdoor environments cannot be separated from the measurements on the comparator track. This verifies that our approach can be applied in normal use. It is only necessary that the scans cover the spectral range of the intensities and that some constraints regarding the scanning geometry are taken into account (Section 4.2).

Our results confirm the findings published in [24,25,26]. According to this, the intensity is a suitable measure to quantify the precision of the distance measurements of a laser scanner. Due to the fact that the pairs between standard deviation ${\hat{\sigma }}_{r}$ and mean intensity $\hat{I}$ show a characteristic exponential behavior, it is reasonable to approximate the data with the functional model [25]:

(6) $${\hat{\sigma }}_{r}=a\cdot {\hat{I}}^{b}+c.$$

For all three scan rates, a model according to Equation (6) was fitted to the data points in Figure 8. This was performed by using a Least-Squares adjustment within the Gauß–Markov model [47,48], where the standard deviations ${\hat{\sigma }}_{r}$ were utilized as observations and the mean intensities $\hat{I}$ as constants. By applying a variance component estimation [47], the stochastic model of the adjustment was adapted to the data allowing for a reasonable estimation of the parameter’s covariance matrix as well as a reasonable filtering of outliers by classical data snooping techniques [47]. For better comparison of the differences between the three scan rates, Figure 8d shows the three estimated models without data points in one plot. Table 4 lists the estimated parameters $\hat{a}$, $\hat{b}$ and $\hat{c}$ of the three models with their estimated standard deviations ${\hat{\sigma }}_{a}$, ${\hat{\sigma }}_{b}$ and ${\hat{\sigma }}_{c}$. Please note that all parameter sets were significant with respect to their estimated covariance matrix. The good approximation is also indicated by the coefficients of determination [49], which were 0.99 for all three models.

As described in Section 3, the noise specifications of the Z + F Profiler 9012A are provided for a standard scan rate of 127 kHz (Table 2). In order to obtain values for the scan rates of 254 kHz, 508 kHz and 1016 kHz, conversion factors have to be used (Table 1) that can be deduced from the law of error propagation for uncorrelated observations with equal precision [47]. In this regard, the ratio between the estimated curves is of interest because it quantifies the noise reduction by selecting a lower scan rate. This ratio can be compared to the conversion factors from theory. The ratio between the models for 1016 kHz and 508 kHz is 1.41 on average, which is exactly $\sqrt{2}$. This is consistent with the ratio of 2.8/2.0 between the related conversion factors and meets the expectation that, due to the halved scan rate, two measurements are averaged. The ratio between the models for 1016 kHz and 254 kHz is 1.81 on average. However, due to the quartered scan rate, a factor of 2.8/1.4=2 was expected.

The lower precision at a scan rate of 254 kHz is most probably caused by the discretization of the distance measurements. The measurements are provided with a resolution of 0.1 mm; however, the representation in the digital signal processing is about 0.17 mm per bit. As a result, the precision at a scan rate of 254 kHz is limited by the discretization. An alternative explanation for the smaller ratio is positive correlations between the measurements that reduce the increase in precision [50]. The improvement of the precision by a factor of 2 only applies to uncorrelated observations.

### 6.2. Investigation of the Close Range Optimization

Regarding the special hardware optimization of the Z + F Profiler 9012A for measurements in the close range (Section 3.3), the data on the comparator track were analyzed in more detail. During the experiments, both white and colored targets were scanned in a range between 0.4 m and 22 m in increments of 0.2 m and 1 m, respectively. These data can be utilized to investigate the measurement noise and the mean intensity as a function of the distance between sensor and object.

As described in Section 6.1, each target scan comprises *i* angular steps of the 2D laser scanner and, thus, *i* pairs of standard deviation ${\hat{\sigma }}_{r}$ and mean intensity $\hat{I}$. These pairs belong to the individual lines in the related point cloud (Figure 7a). For better interpretation, one of these pairs was determined to be representative for the specific target scan. This was realized by choosing the angular step of each target scan containing the median of all standard deviations along with its mean intensity.

**Figure 9:**
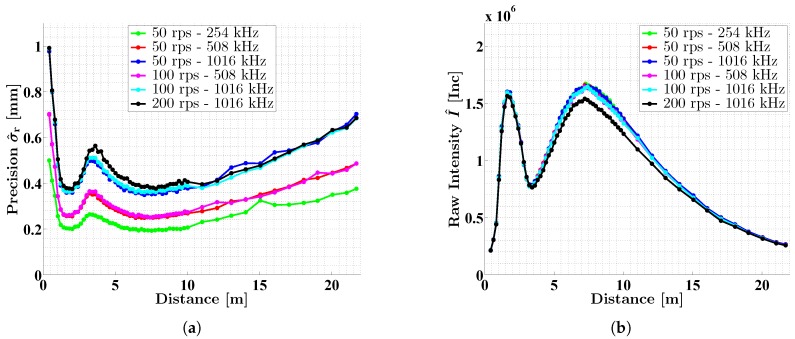
Precision $\hat{\sigma_{r}}$ (**a**) and intensity $\hat{I}$ (**b**) of the distance measurements of the Z + F Profiler 9012A as a function of distance between sensor and object. The scans were carried out on a white planar target with all six settings of the 2D laser scanner (Table 1).

Figure 9a shows the representative standard deviations and mean intensities as a function of the distance between laser scanner and object for the scans on the white target from experiment 1. The individual curves correspond to the different settings of the Z + F Profiler 9012A (Table 1). Clearly, all curves follow a similar pattern. The precision of the distance measurements is increased directly in front of the 2D laser scanner, but quickly drops to a local minimum at about 2 m. Following this, the precision worsens in the range between 2 m and 5 m with a maximum value at approximately 3.5 m. For distances larger than 5 m, the curves sink to the global minimum around 8 m before increasing steadily up to the end of the investigated range. It is likely that this behavior continues beyond 22 m due to the signal attenuation with the squared distance [1,2,44].

According to Figure 9a, the hardware-based close range optimization of the Z + F Profiler 9012A has its optimum at about 2 m, producing a noise that is of the same magnitude as the noise at about 8 m. Especially at 3.5 m, one has to be aware of increased noise, which cannot be completely compensated by the close range optimization. The special behavior of the curves in the range below 5 m is caused by the intensity of the backscattered laser light. This is proved by Figure 9b, which shows the related mean intensities of Figure 9a as a function of distance. The noise and intensity curves behave in exactly the opposite way. Furthermore, the intensity curves in Figure 9b are equivalent to the simulated blue curve in Figure 2a. This demonstrates that theory and reality agree well. Moreover, these results verify that the intensity-based stochastic models are also valid in the range below 5 m since the data from the close range also contribute to the determination of the stochastic models in Figure 8.

As can be seen from Figure 9a, curves belonging to the same scan rate overlap in good accordance. In addition, the factors of 1.4 and 1.8 between the scan rates (Section 6.1) can be identified again. This emphasizes that both the intensity and the scan rate are the decisive factors for the distance precision. According to this, an intensity-based stochastic model only applies to a certain scan rate. Please note that only the scan rate matters and not how it is generated. In the case of Z + F Profiler 9012A, for instance, a scan rate of 1016 kHz can be generated by three different combinations of mirror speed and point resolution (Table 1). However, the black curves in Figure 9 (mirror speed: 200 rps, scan rate: 1016 kHz) are slightly different in this respect since they do not completely overlap with the blue and cyan curves. In Figure 9a, the black curves are a bit higher, in Figure 9b a bit deeper in the middle part. However, this is consistent for the relationship between precision and intensity. Thus, the stochastic model from Figure 8a is not affected. An explanation for this special behavior could not be identified so far. Probably, there is a connection to the high rotational speed of 200 rps of the deflecting mirror.

**Figure 10:**
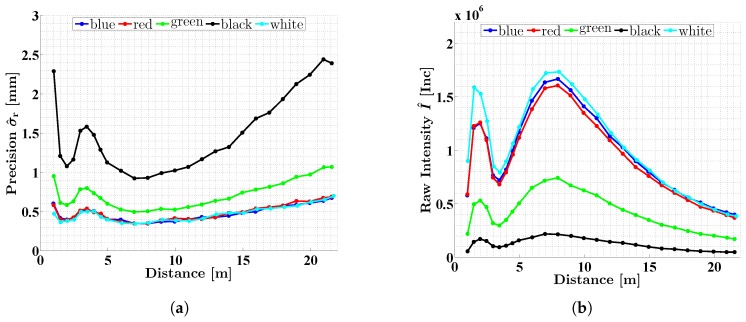
Precision $\hat{\sigma_{r}}$ (**a**) and intensity $\hat{I}$ (**b**) of the distance measurements of the Z + F Profiler 9012A as a function of distance between sensor and object. The scans were carried out on colored planar targets (mirror speed: 50 rps, scan rate: 1016 kHz).

The distance-related investigations were also carried out for the colored targets. The results are shown in Figure 10. All curves refer to a mirror speed of 50 rps and a scan rate of 1016 kHz. For better interpretation, the corresponding curves from Figure 9 are added to Figure 10. The characteristic shape of the curves as observed for the white target can also be found for the colored targets. However, the level of precision is very different. Whereas the precision of the white, blue and red curves is almost the same, the precision of the green and black curve is considerably worse (Figure 10a) due to the lower intensity of the laser light (Figure 10b). The characteristics of the curves are more distinctive with decreasing reflectivity of the surface. For example, the noise of the black curve increases by 0.5 mm between 2 m and 3.5 m, whereas the difference for the white target is only 0.15 mm. The precision of ${\hat{\sigma }}_{r}=1.6\;\mathrm{mm}$ for the black target at 3.5 m seems to be small, but this means that 99.7 % (i.e., $\pm \;3\;{\hat{\sigma }}_{r}$) of the measurements are scattered over an interval of almost 1 cm. For precise measurements of dark asphalt surfaces, this is highly relevant. In practice, therefore, it is advisable to mount the Z + F Profiler 9012A not in a distance of 3.5 m to the road surface, but rather 2 m or 5 m.

## 7. Conclusions

Within this work, the intensity-dependent stochastic distance characteristics of 2D laser scanners with special focus on the close range were addressed. In the close range, divergent incident laser light can lead to a reduction of the signal input power deteriorating both the backscattered intensity and the precision of the distance measurements. This is highly relevant for kinematic applications when scanning, e.g., the road surface or tunnel walls. In particular, the 2D laser scanner Z + F Profiler 9012A was investigated. This sensor is of special interest since it is equipped with a hardware optimization that partially compensates for the problems in the close range.

For analyzing the intensity-dependent distance precision, we proposed a new approach for 2D laser scanners since previous research [24,25,26] only focused on methods for 3D laser scanners that are not applicable to 2D laser scanners. The new approach is based on the static scanning of surfaces with different backscatter. The large number of overlapping scanning profiles is used to analyze the noise as a function of the backscattered intensity. Our method is beneficial because the laser scanner is operated in its normal measurement mode, no model assumptions for the scanned surface are made and no sophisticated equipment is required, making the approach feasible for operators in normal use. The only requirement is that certain precautions regarding the scanning geometry should be taken into account. Moreover, the scans should cover the intensity range as much as possible.

The approach is used to derive three stochastic models for the three scan rates of the Z + F Profiler 9012A, clearly indicating the dependency between intensity, scan rate and distance precision [24]. Our method also allows for a detailed analysis of the close range. Regarding this, a lower precision caused by a lower intensity could be verified for the range < 5 m. The special hardware optimization of the Z + F Profiler 9012A partially compensates for this. This optimization was found to work best at about 2 m, but operators must still be critical of measurements, especially at distances below 1 m and at about 3.5 m. This is useful information when mounting the laser scanner to a mobile platform. However, the derived stochastic models remain valid also in the range below 5 m. These models are relevant, e.g., for quality assurance and deformation analysis. In addition, they could be used in the system calibration leading to better estimates for the calibration parameters [43,45].

This article contributes to a better understanding of the quality of the Z + F Profiler 9012A and laser scanners in general. Future work should analyze the accuracy of the Z + F Profiler 9012A. For this purpose, the interferometric comparator track in our laboratory could be used. Such investigations could be oriented towards the works of [2,13,32].

## Figures and Tables

**Table 1:** Different settings of the Z + F Profiler 9012A (mirror speed, number of points per scanning profile) along with the factors for converting the noise specifications for the standard scan rate of 127 kHz (Table 2) to the actual scan rates of 254 kHz, 508 kHz or 1016 kHz (according to [27]).

Mirror Speed	200 rps	100 rps	50 rps
Points/360${}^{\circ }$	Scan rate/Noise factor	Scan rate/Noise factor	Scan rate/Noise factor
20,480	—	—	1016 kHz/× 2.8
10,240	—	1016 kHz/× 2.8	508 kHz/× 2.0
5120	1016 kHz/× 2.8	508 kHz/× 2.0	254 kHz/× 1.4

**Table 2:** Specified standard deviations $\sigma_{r}$ of the distance measurements for a standard scan rate of 127 kHz for different distances as well as reflectivities of the surface. The values are stated for both Z + F Profiler 9012/9012A (without/with close range optimization) and have to be converted to the actual scan rates of 254 kHz, 508 kHz or 1016 kHz by using the factors from Table 1 (according to [27]).

Distance Noise	Z + F Profiler 9012 | 9012A	Z + F Profiler 9012 | 9012A	Z + F Profiler 9012 | 9012A
Target Distance	White (80 %)	Grey (37 %)	Black (14 %)
1 $\sigma_{r}$ @ 0.5 m	0.5 mm | 0.5 mm	0.8 mm | 0.8 mm	1.3 mm | 1.3 mm
1 $\sigma_{r}$ @ 1 m	0.5 mm | 0.3 mm	0.6 mm | 0.4 mm	1.0 mm | 0.8 mm
1 $\sigma_{r}$ @ 2 m	0.3 mm | 0.2 mm	0.5 mm | 0.3 mm	0.8 mm | 0.4 mm
1 $\sigma_{r}$ @ 5 m	0.3 mm | 0.2 mm	0.4 mm | 0.3 mm	0.6 mm | 0.5 mm
1 $\sigma_{r}$ @ 10 m	0.2 mm | 0.2 mm	0.3 mm | 0.3 mm	0.5 mm | 0.5 mm
1 $\sigma_{r}$ @ 25 m	0.4 mm | 0.4 mm	0.6 mm | 0.6 mm	1.1 mm | 1.1 mm
1 $\sigma_{r}$ @ 50 m	0.9 mm | 0.9 mm	1.4 mm | 1.4 mm	3.1 mm | 3.1 mm

**Table 3:** Data basis for the determination of intensity-based stochastic models for all three scan rates of the Z + F Profiler 9012A (i.e., 254 kHz, 508 kHz and 1016 kHz).

Scan Rate	Mirror Speed	Experiments	Pairs ${\hat{\sigma }}_{r}$/$\hat{I}$
254 kHz (= 254,000 points/s)	50 rps	1, 4	# 47969
508 kHz (= 508,000 points/s)	50 rps, 100 rps	1, 4	# 81484
1016 kHz (= 1,016,000 points/s)	50 rps, 100 rps, 200 rps	1, 2, 3	# 141858

**Table 4:** Estimated parameters of the intensity-based stochastic models for the Z + F Profiler 9012A.

Scan Rate	$\hat{a}$ $\left[\frac{m}{\mathrm{Inc}}\right]$	${\hat{\sigma }}_{a}$	$\hat{b}$ [−]	${\hat{\sigma }}_{b}$	$\hat{c}$ [m]	${\hat{\sigma }}_{c}$
**254 kHz**	7.09896	0.03902736	−0.80377	0.00055374	0.00014	0.00000030
**508 kHz**	8.21610	0.02611121	−0.78192	0.00031518	0.00015	0.00000026
**1016 kHz**	15.67256	0.03579010	−0.81170	0.00022396	0.00024	0.00000024

## Abbreviations

APD	Avalanche Photo Diode
GNSS	Global Navigation Satellite System
IMU	Inertial Measurement Unit
kHz	kilohertz
rps	rotations per second
RTK	Real-Time Kinematic
Z + F	Zoller + Fröhlich
